# Statistical shape modeling of multi-organ anatomies with shared boundaries

**DOI:** 10.3389/fbioe.2022.1078800

**Published:** 2023-01-12

**Authors:** Krithika Iyer, Alan Morris, Brian Zenger, Karthik Karanth, Nawazish Khan, Benjamin A. Orkild, Oleksandre Korshak, Shireen Elhabian

**Affiliations:** ^1^ University of Utah, School of Computing, Salt Lake City, UT, United States; ^2^ University of Utah, Scientific Computing and Imaging Institute, Salt Lake City, UT, United States; ^3^ University of Utah School of Medicine, Salt Lake City, UT, United States; ^4^ University of Utah, Department of Biomedical Engineering, Salt Lake City, UT, United States

**Keywords:** statistical shape modeling, biventricular cardiac MRI, particle-based shape modeling, interventricular septum, shared boundaries

## Abstract

**Introduction:** Statistical shape modeling (SSM) is a valuable and powerful tool to generate a detailed representation of complex anatomy that enables quantitative analysis of shapes and their variations. SSM applies mathematics, statistics, and computing to parse the shape into some quantitative representation (such as correspondence points or landmarks) which can be used to study the covariance patterns of the shapes and answer various questions about the anatomical variations across the population. Complex anatomical structures have many diverse parts with varying interactions or intricate architecture. For example, the heart is a four-chambered organ with several shared boundaries between chambers. Subtle shape changes within the shared boundaries of the heart can indicate potential pathologic changes such as right ventricular overload. Early detection and robust quantification could provide insight into ideal treatment techniques and intervention timing. However, existing SSM methods do not explicitly handle shared boundaries which aid in a better understanding of the anatomy of interest. If shared boundaries are not explicitly modeled, it restricts the capability of the shape model to identify the pathological shape changes occurring at the shared boundary. Hence, this paper presents a general and flexible data-driven approach for building statistical shape models of multi-organ anatomies with shared boundaries that explicitly model contact surfaces.

**Methods:** This work focuses on particle-based shape modeling (PSM), a state-of-art SSM approach for building shape models by optimizing the position of correspondence particles. The proposed PSM strategy for handling shared boundaries entails (a) detecting and extracting the shared boundary surface and contour (outline of the surface mesh/isoline) of the meshes of the two organs, (b) followed by a formulation for a correspondence-based optimization algorithm to build a multi-organ anatomy statistical shape model that captures morphological and alignment changes of individual organs and their shared boundary surfaces throughout the population.

**Results:** We demonstrate the shared boundary pipeline using a toy dataset of parameterized shapes and a clinical dataset of the biventricular heart models. The shared boundary model for the cardiac biventricular data achieves consistent parameterization of the shared surface (interventricular septum) and identifies the curvature of the interventricular septum as pathological shape differences.

## 1 Introduction

It has long been recognized in the anatomical sciences that the human body exhibits various morphological patterns and configurations, referred to as *anatomical variation*. Variations are prevalent throughout the body and may cause or be a factor resulting in a significant medical condition. To carry out a wide range of surgical and other medical procedures and therapeutic modalities, it is essential to have a thorough understanding of natural anatomical variation (Smith, 2021). Primitively, the morphometric variations of anatomies were commonly reported through observational studies that inspect large numbers of cadavers, and medical images (Alraddadi, 2021). By permitting in-depth, non-invasive investigation of the human body, recent advancements in medical imaging, such as Magnetic Resonance Imaging (MRI) and computed tomography (CT) scans, have significantly increased the understanding of the complexity of human anatomy. Owing to the growing interest in studying anatomical variations, statistical shape modeling (SSM) has emerged as an essential computational tool that discovers significant shape parameters directly from medical data (such as MRI and CT scans) that can fully quantitatively describe complex anatomy in the context of a population.

Statistical shape models are used to perform wide range of tasks in biomedical research ranging from visualizing organs (Orkild et al., 2022), bones (Lenz et al., 2021), and tumors (Krol et al., 2013), to aiding surgical planning (Borghi et al., 2020), monitoring disease progression (Uetani et al., 2015; Faber et al., 2020), and implant design (Goparaju et al., 2022). Shapes can be represented using an implicit (deformation fields (Durrleman et al., 2014), level set methods (Samson et al., 2000)) or explicit (set of ordered landmarks/points) representation. For explicit representations, points of the same anatomical position must be established consistently across shape populations to enable shape comparisons and obtain population-level shape statistics in an ensemble of shapes. These points are called *correspondences*. Explicit parameterization, such as correspondence points, is one of the most popular techniques used to represent shapes because of their simplicity and ability to represent multiple objects easily (Cerrolaza et al., 2019). Hence, in this work, we focus on point distribution models (PDM), which are a dense set of correspondences for shape representation. Multiple methods for correspondence generation have been proposed, which include non-optimized landmark estimation, parametric and non-parametric correspondence optimization. Non-optimized methods entail manually annotating the reference shape and warping the annotated landmarks on the population data using image-based or shape-based registration (McInerney and Terzopoulos, 1996; Paulsen et al., 2002; Heitz et al., 2005). Such non-optimized methods employ hard surface constraints to distribute points on a shape. Parametric methods use fixed geometrical basis (e.g., spheres (Styner et al., 2006)) to parameterize objects and generate correspondences. Correspondence models obtained using manual or parametric techniques are not optimal and can be incapable of handling complex shapes as the expressivity of the models is limited by choice of the fixed geometrical basis or template. On the other hand, non-parametric automatic methods provide a robust and general framework as they generate PDMs without relying on a specific geometric basis. Methods that follow a group-wise non-parametric approach find the correspondence by considering the variability of the entire cohort in the optimization process (e.g., particle-based optimization (Cates et al., 2017) and Minimum Description Length - MDL (Davies, 2002)).

Traditional SSM methods started by creating single-organ anatomy models particular to an organ or disease. However, the human body comprises intricate organs and systems that are physically, functionally, and spatially interrelated (Sanfilippo et al., 1990; Cates et al., 2014; Marrouche et al., 2014). For example, the hip joint is a ball and socket joint, with articular cartilage covering the articulating surfaces of the femur and pelvis. Similarly, the sacroiliac joint is a diarthrodial auricular joint between the sacrum and the ilium that allows bipedal movement. Due to the nature of these joints, subject-specific bone and cartilage anatomy drive the contact mechanics of the joint. Even subtle variations in anatomy may result in abnormal cartilage contact mechanics and lead to osteoarthritis (Dreyfuss et al., 2004; Andriacchi et al., 2009). Simultaneous quantification of the shape of the cartilage surface and the shared subchondral bone surface may help elucidate the joint’s complex, dynamic articulation and diagnose biomechanical pathologies (Li et al., 2013; Jesse et al., 2017; Postacchini et al., 2017). Another example of interconnected anatomy is the heart, a four-chambered organ with several shared boundaries between chambers. Coordinated and efficient contraction of the chambers of the heart is necessary to adequately perfuse end organs throughout the body. Subtle shape changes within these shared boundaries of the heart can indicate potential pathological changes that lead to uncoordinated contraction and poor end-organ perfusion. Thorough examination and understanding of various interconnected organ systems are paramount to diagnosing and providing prompt therapeutic support (Bartsch et al., 2015). Hence, the attention of recent computational anatomy research has shifted from single-organ to multi-organ models (Cerrolaza et al., 2019). Multi-organ shape models perform joint statistical shape analysis to quantify meaningful shape variations and contextual information when studying the group differences and identifying the shape differences occurring due to a particular pathology affecting multiple interacting organs.

The group-wise SSM approaches mentioned previously have been extended to model multi-organ anatomies. These approaches either parameterize each object separately, sacrificing anatomical integrity (Cerrolaza et al., 2019), or minimize the combined cost function to generate correspondences assuming a global statistical model (Cates et al., 2008; Durrleman et al., 2014). However, these multi-organ models often fail to incorporate nuanced interactions such as shared surfaces (cartilage of the hip joint or the sacroiliac joint or interventricular septum of the heart) between multiple anatomies that can reveal critical features that might not be observable when the individual organs are modeled independently.

To address this issue, we propose a new shape modeling workflow that entails a method for extracting shared boundary surfaces and a correspondence-based optimization scheme to parameterize multi-organ anatomies and their shared surfaces consistently. We demonstrate the entire workflow using a cardiac biventricular dataset, where we model the right ventricle (RV), left ventricle wall (LVW), and interventricular septum (IVS). We build upon the group-wise, non-parametric particle-based optimization method proposed by Cates et al., (Cates et al., 2007; Cates et al., 2008; Cates et al., 2017), to generate PDM and modify the framework to support multi-organ anatomies with shared boundaries.

The preliminary results of this work have been published in a workshop paper (Iyer et al., 2022). Here we significantly expand this work as follows.

1. Detailed experiments to convey the proof-of-concept with a synthetically generated parameterized set of shapes (the peanut dataset).
2. Study the necessity and effectiveness of modeling the shared boundary by comparing the modes of variations and group differences inferred using the shared boundary model of the biventricular anatomy with multi-organ shape models without explicitly shared boundary parameterization.
3. Perform multi-level analysis for the multi-organ shape models to disentangle pose from shape variations.
4. Perform ablation experiments to study the effect of class imbalance on the shape model generation process.

## 2 Methods

With a PDM, a shape can be represented as a vector that contains the coordinates of all of its surface correspondences. This concept can be broadened to encompass an ensemble of shapes, allowing for the representation of all shapes in a high-dimensional vector space, the shape space, and aiding in the investigation of how shapes are distributed to identify geometric variation patterns between the structures of interest. Statistical shape models are, in their most basic form, concise mathematical representations of objects that successfully parameterize every shape in the shape space. Herein we leverage the particle-based shape modeling (PSM) approach (Cates et al., 2007; Cates et al., 2017) for automatically constructing PDMs by optimizing point (or particle) distributions over a cohort of shapes using an entropy-based optimization method.

There are two essential considerations for modeling interconnected anatomical structures with surface openings and shared boundaries. First, it is necessary to explicitly characterize the statistics of the exterior (contour) and the interior of the shared surface to build statistical shape models that are aware of the interactions of the organs. This requires a consistent point distribution on the shared boundary across the multi-organ anatomies. To meet these needs, we develop methods for detecting and extracting shared boundaries and their edges (i.e., contour information) from multi-organ anatomies (see Section 2.2). Second, we need to optimize a PDM that includes joint statistics of the multi-organ anatomies, shared boundary interior, and contour. The PSM method proposed by Cates et al., which forms the foundation of our proposed method, uses a system of interacting particles with mutually repelling forces that learn the most compact statistical descriptors of the anatomy (Cates et al., 2008; Cates et al., 2017). For consistent parameterization on the shared boundary, we modify the surface sampling objective of the PSM method to accommodate the interaction between the anatomies and the shared surface. A brief overview of the PSM entropy optimization method for single anatomy is provided in Section 2.1 and the proposed surface cost function modifications for multi-organ anatomies with shared boundary surfaces is provided in Section 2.3.

### 2.1 Background: Particle-based shape modeling (PSM)

PDMs offer a framework for quantifying statistical relations between several factors representing the morphology of anatomy (Cootes et al., 1995). Using principal component analysis (PCA) on PDMs, it is possible to quantify population-level morphological variations. Therefore, an anatomical mapping across all anatomical (shape) samples in the given cohort should be established to obtain meaningful statistical shape variations. PSM offers a data-driven approach to establishing such mapping by establishing dense surface correspondences without needing an initial atlas or template. PSM learns the shape parameters by optimizing the position of a system of interacting particles such that the shape model can completely describe the variability of the population using the most compact statistical model that still preserves geometrical accuracy. In this section, we briefly describe the PSM method proposed by Cates et al., (Cates et al., 2007; Cates et al., 2008), that will be later modified in Section 2.3 to capture meaningful and consistent shape models for multi-organ anatomies with shared boundaries.

Consider a cohort of shapes $S=\left\{z_{1},z_{2},\ldots ,z_{N}\right\}$ of *N* surfaces, each with its set of *M* corresponding particles $z_{n}=\left[x_{1},x_{2},\ldots ,x_{M}\right]\in R^{dM}$ where each particle $x_{m}\in R^{d}$ lives in *d*−dimensional Cartesian (i.e., configuration) space. This work uses surface meshes where *d* = 3. The ordering of the particles implies correspondence among shapes. Each correspondence particle is constrained to lie on the shape’s surface. Collectively, the set of *M* particles is known as the *configuration*, and the space of all possible configurations is known as the *configuration space*. The particle positions are samples (i.e., realizations) of a random variable $X\in R^{d}$ in the configuration space with an associated probability distribution function (PDF) *p* (**X = x**). Each configuration of *M* particles can be mapped to a single point in *dM*−dimensional *shape space* by concatenating the correspondence coordinate positions into a single vector **z**_*n*_. The vector **z**_*n*_ is modeled as an instance of random variable **Z** in the shape space with PDF *p* (**Z = z**) assuming shapes are Gaussian distributed in the shape space, i.e., Z∼N(μ,Σ) . The optimization proposed by Cates et al., (Cates et al., 2007; Cates et al., 2017), to establish correspondence minimizes the energy function

$$Q=H\left(Z\right)-\overset{N}{\underset{n=1}{\sum }}H\left(X_{n}\right)$$ (1)

where *H* is an estimation of differential entropy. The differential entropy of *p*(**X**) is given as

$$H\left(X\right)=-\int_{S}p\left(X\right)\log p\left(X\right)dx=-E\left\{\log p\left(X\right)\right\}\approx -\frac{1}{M}\overset{M}{\underset{m=1}{\sum }}\log p\left(x_{m}\right)$$ (2)

where *H*(**X**) is by calculating by estimating the density function *p*(**X**) using a nonparametric, Parzen windowing estimation method with the help of the particles. The entropy in the shape space for the Gaussian distribution is calculated as $H\left(Z\right)=\frac{1}{2}\log|\Sigma |$ . More details regarding entropy terms can be found in (Cates et al., 2007; Cates et al., 2017). Gradient descent is used to minimize the cost function *Q*. Minimization of the first term in *Q* from Eq 1 produces a compact distribution of samples in shape space and encourages particles to be in correspondence across shapes. The second term seeks uniformly-distributed correspondence positions on the shape surfaces for a geometrically accurate shape representation (Cates et al., 2007; Cates et al., 2017). The negative gradient $-\frac{\partial H\left(Z\right)}{\partial Z}$ provides an update vector for the entire particle system, which is computed once per iteration, i.e., assuming lagging shape statistics for optimization stability. The individual shape-based updates $\frac{\partial H\left(X_{n}\right)}{\partial X_{n}}$ are combined with the negative gradient term to provide the update for each particle. Further details regarding the optimization and gradient updates can be found in (Cates et al., 2007; Cates et al., 2017).

A common coordinate system must be used to perform statistical analysis of the PDMs. Hence, the PDMs are built by factoring out global scaling, rotation, and translation. Typically the input shape data (volume segmentations or surface meshes) are aligned as a pre-processing step using iterative closed point alignment (Arun et al., 1987; Besl and McKay, 1992) that registers the shapes to an unbiased coordinate system by iteratively minimizing the pairwise least squares difference between the individual shapes and a reference shape. Once the similarity transformations have been removed, the statistical shape model can be easily constructed and analyzed.

### 2.2 Shared boundary extraction

**FIGURE 1:**
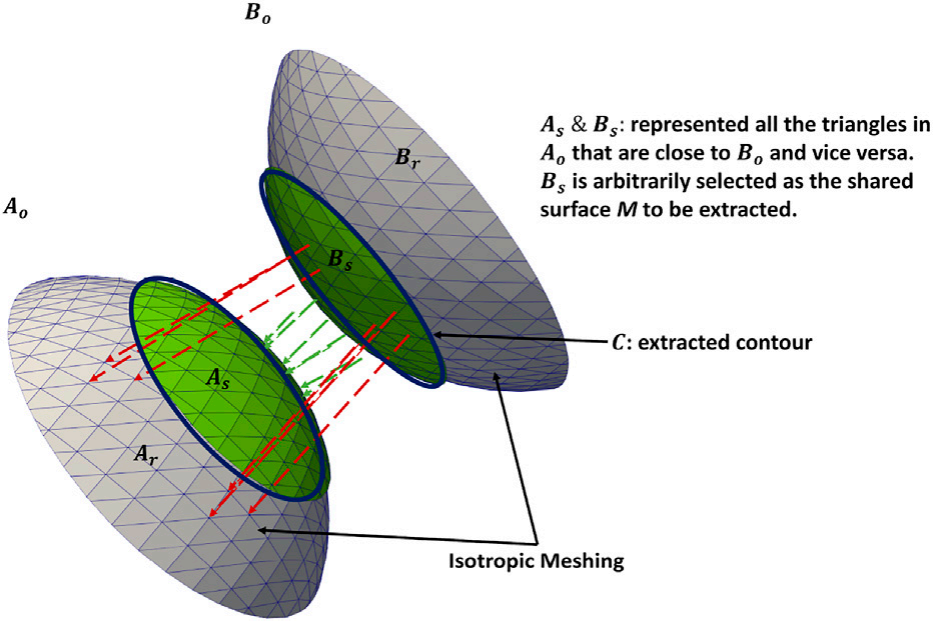
Extracting shared boundary between two meshes. The regions in green have Euclidean distances that fall within the threshold and are extracted as a shared boundary as per step 2. The green arrows show the distances within the threshold for the vertices included in the shared boundary. The red arrows show distances greater than the threshold for the vertices excluded from the shared boundary. The contour is extracted from the green region as per step 4. Note: the meshes are farther apart, and the threshold is larger for visualization purposes.

To demonstrate the shared boundary extraction pipeline, consider two adjoining organs *A* and *B*, with a shared boundary (Figure 1). The steps for shared boundary extraction entail the following.

**FIGURE 2:**
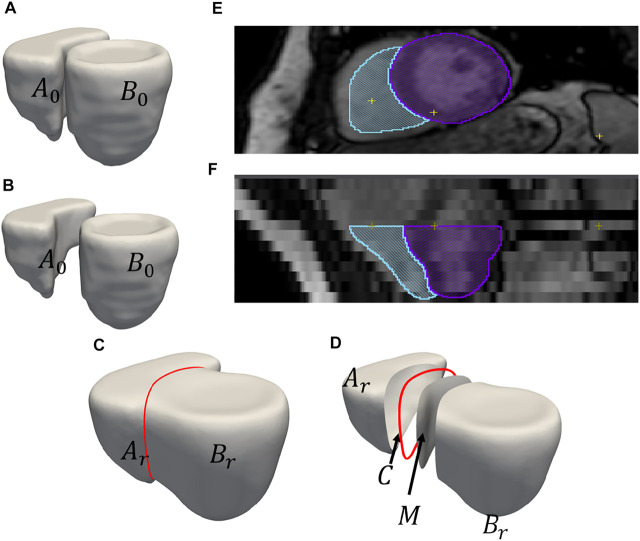
An example of output obtained after shared boundary extraction. Meshes representing **(A)** RV and LVW show that they have a shared boundary surface, and **(B)** RV and LVW meshes are pried apart. The meshes and contour obtained after shared boundary extraction **(C)** RV, LVW, shared surface and contour **(D)** all outputs pried apart for visualization. The red color indicates the contour. The image shows 2D slices of the endocardial segmentation for the RV (blue) and epicardial segmentation for the LV (violet) at end-diastole in the **(E)** axial view and **(F)** coronal view.

1. **Isotropic Explicit Re-meshing:** This generates a new mesh triangulation that conforms to the original data but contains more uniformly sized triangles. Re-meshing improves the quality of the mesh while preserving the original geometrical features. Re-meshing also has the benefit of ensuring equivalent average edge lengths across the two shapes, which is useful in ensuing steps (Valette et al., 2008).
2. **Extracting Shared Boundary:** In this step, we pass the two adjacent organs to the extraction tool that then outputs three new shapes, two of which correspond to the original shapes (without the shared boundary surface) and one for the shared boundary. To look into the overview of the algorithmic steps involved in the extraction tool, let us designate the original meshes of the adjoining organs as *A*_*o*_ and *B*_*o*_ (Figures 2A, B) then:
a. Find all the triangles in *A*_*o*_ that are close to *B*_*o*_ and construct a mesh with these triangles called *A*_*s*_. A triangle with vertices (*v*_0_, *v*_1_, *v*_2_) is considered close to another mesh if the shortest Euclidean distance between all three vertices and the other mesh is less than a specific threshold. The threshold must be experimentally tuned for the data to ensure the extracted shared surfaces are clinically relevant.
b. We similarly find all the triangles in *B*_*o*_ that are close to *A*_*o*_ and designate this mesh as *B*_*s*_.
c. Find the remainder of the mesh in *A*_*o*_ after removing the triangles in *A*_*s*_ and designate this as *A*_*r*_. Similarly, we designate the remainder of the mesh in *B*_*o*_ after removing the triangles in *B*_*s*_ as *B*_*r*_.
d. Since, *A*_*s*_ = *B*_*s*_, we arbitrarily designate *B*_*s*_ as the shared surface *M*
e. Copy all the points on the boundary loop of *A*_*r*_ to the boundary loop of *M* and return three new shapes *A*_*r*_, *M*, and *B*_*r*_ (Figure 2C).
3. **Laplacian Smoothing:** At this point, the resulting triangulation typically contains jagged edges. We apply Laplacian smoothing to correct for this (Field, 1988). Laplacian smoothing reduces noisy edges/artifacts found on the mesh surface with minimal changes to its shape. This results in cells with better shapes and evenly distributed vertices.
4. **Extract Contour:** The boundary loop of the shared surface *M* is computed using LibIGL *boundary_loop* tool (Jacobson and Panozzo, 2018) and designate this contour as *C* (Figure 2D).

The input consisting of two adjoining organs *A*_*o*_ and *B*_*o*_ with a shared surface has been converted into input with four separate parts, the organs *A*_*r*_ and *B*_*r*_, the shared surface *M*, and the contour *C* using the pipeline (Figure 2D; Figure 1).

### 2.3 Particle-based shape modeling with shared boundaries

A shape model built for multi-organ anatomies with shared boundaries requires a shape model that faithfully captures the joint statistics of all the interacting organs while consistently representing the individual organs. In order to capture the joint statistics of the multi-organ system, particle-based optimization should be capable of handling multi-organ anatomies. The optimization set up in Eq 1 was extended for multiple organs in (Cates et al., 2008). From the PSM formulation for single anatomies mentioned in the Section 2.1, it is important to note that *p* (**x**_*m*_) in Eq 2 was estimated from the particle position using non-parametric kernel density estimation method (Cates et al., 2007; Cates et al., 2017). This results in a set of points on the surface that repel each other with Gaussian-weighted forces. For multi-organ anatomy, the optimization is extended so that if one organ has a distinct unconnected surface, the spatial interactions between particles on different organs are decoupled, and particles are constrained to lie on a single organ (surface). This is enforced by considering the entropy of the correspondences in the configuration space (second term in Eq. 1) of each organ of each anatomy separately. This separation ensures that the particles are uniformly distributed on each organ independently. At the same time, the covariance **Σ** of the random variable **Z** in the space includes all particle positions across the multiple organs. This ensures the optimization takes place on the multi-organ shape space and the shape statistics remain coupled (Cates et al., 2008) resulting in an overall compact model and particles in correspondence on all organs and across anatomies. For *K* organs in anatomy, the cost function as in (10) is

$$Q=H\left(Z\right)-\overset{K}{\underset{k=1}{\sum }}\left[\overset{N}{\underset{n=1}{\sum }}H\left(X_{n}^{k}\right)\right]$$ (3)

where $X_{n}^{k}$ represents the particle random variable associated with the *n* ^th^ anatomy (or subject) and the *k* ^th^ organ.

From Eq. 3, the second term, representing the sampling objective, is summed over all the shape samples. The sampling is restricted to the particles within the individual organs. As a result, when two organs have a shared boundary and sampling is done independently, it raises concerns about the statistics captured for the shared boundary surface using two particle systems. As there is no explicit representation of the common shared boundary, when the statistical analysis is performed for anatomies with shared boundaries, there is no mechanism to prevent the particles from penetrating other organs while studying the modes of variations. Such shape models with poorly parameterized anatomies and their interactions lead to a clinically incorrect statistical representation of morphological variations and observation. Hence, the shared boundary has to be explicitly parameterized into two parts - the interior (shared boundary surface) and exterior (shared boundary contour) of the surface. The extracted shared boundary has to be modeled as a separate entity from the two organs to avoid capturing the same statistics from multiple particle systems of the multi-organ anatomy. Another important consideration is that we need to ensure that the particles do not clutter around the edges of the organs and the shared boundary surface and contour. Hence, the interaction between the organs and the extracted shared boundary surface and contour has to be introduced during the optimization process. The sampling objective needs to be modified to introduce the interaction so that the particles on the shared boundary contour repel the particles of other organs to present each organ faithfully. This will result in a buffer distance between particles of the multiple organs leading to a uniform correspondence model and discouraging particles from moving into other organs during the representation of morphological variations.

The proposed objective function is:

$$Q=H\left(Z\right)-\left[\underset{k\in \left(A_{r},M,B_{r}\right)}{\sum }\overset{N}{\underset{n=1}{\sum }}H\left(X_{n}^{k}X_{n}^{C}\right)+\overset{N}{\underset{n=1}{\sum }}H\left(X_{n}^{C}\right)\right]$$ (4)

where **X** ^*C*^ is the matrix of particle positions located in the contour. Effectively, this means that all the particles on the *A*_*r*_, *M* and *B*_*r*_ are repelled by particles on the contour *C*. Similar to the original PSM formulation mentioned in Section 2.1, the cost function Q from Eq 4 is minimized using gradient descent. As a general assumption, the surface area and circumference of the shared boundary surface are smaller than the anatomy’s individual organs. The number of particles required to describe the shared boundary and contour is much lower than the number of particles required to describe the organs. Hence, we do not change the sampling objective for the contour ( $\sum_{n=1}^{N}H\left(X_{n}^{C}\right)$ term in Eq 4. This is because the large magnitude of gradients from more particles of the meshes could cause the particles on the contour to swap places. Since there is only one degree of freedom on a contour, it is almost impossible to recover from this situation.

## 3 Dataset and shape modeling

### 3.1 Synthetic peanut dataset

**FIGURE 3:**
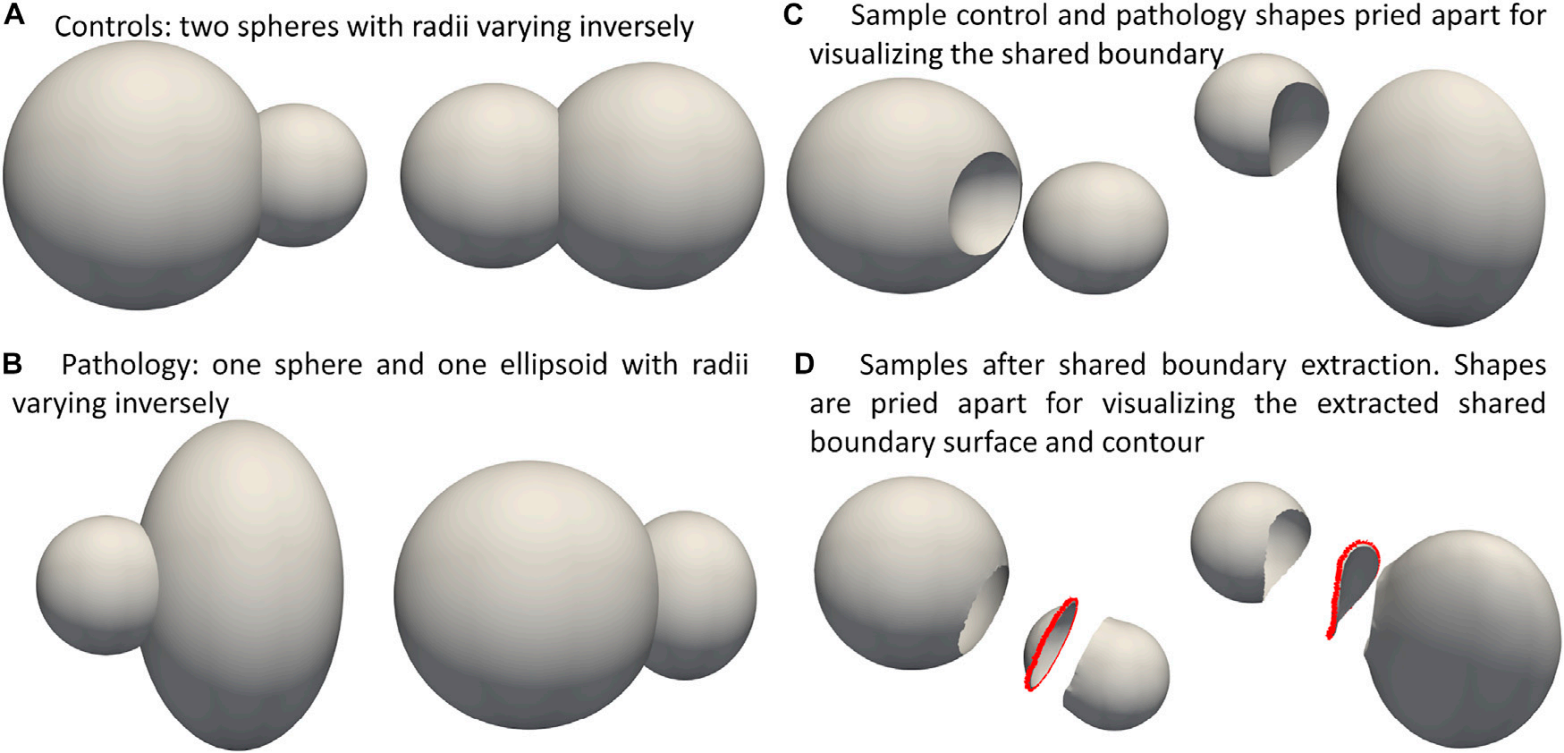
Synthetic Peanut Dataset: Surface meshes representing the two groups included in the peanut dataset- **(A)** Controls group: two spheres with varying radii, **(B)** pathalogical changes are emulated by changing one of the spheres to ellipsoid. **(C)** Samples from the controls and pathalogy groups show casing the shared boundary surface, **(D)** sample outputs obtained after extracting the shared boundary surface and contour. The meshes are pried apart for visualization.

We demonstrate the proposed pipeline for shared boundary extraction and optimization of models with shared surfaces by considering a synthetic peanut dataset. Each sample in this dataset consists of surface meshes of two spheres, but one of the spheres is subtracted from the other. There exists a shared surface between the two spheres (Figure 3C). The radii of the two spheres vary inversely, i.e., as one gets bigger, the other gets smaller (Figure 3A). Pathological shape changes are emulated by converting one of the spheres to ellipsoids where the radii in the *y* and *z* direction are varied (Figure 3B). The dataset is balanced and consists of 15 controls (i.e., where both the shapes are spheres) and 15 pathology (i.e., where one of the shapes is an ellipsoid.) All the shapes are alignment and are centered at $\left[0,0,0\right]$ during the data generation process.

### 3.2 Cardiac biventricular dataset

We evaluated our method on a real world cardiac biventricular dataset, comparing how well the resulting correspondence model captures variability in shape for cardiovascular clinic patients and healthy volunteer groups. The dataset consists of MRIs of six healthy volunteers and 23 patients treated at a cardiovascular clinic. In the patient group, tricuspid regurgitation was secondary to pulmonary hypertension in one patient; congestive heart failure (CHF) in 10 patients; and other causes (atrial fibrillation, pacemaker lead injury, pacemaker implantation, congenital heart disease) in 12 patients. The healthy volunteers had no diagnosis of cardiac disease and no cardiovascular risk factors. The 23 patients were retrospectively identified from the University of Utah medical data warehouse after verification of the patient charts. Healthy volunteer images were obtained during a previous study at Weill-Cornell Medical College, after IRB approval (Orkild et al., 2022). These studies involving human participants were reviewed and approved by the University of Utah Internal Review Board committee.

RV and LVW shapes were generated from manual segmentations performed on reconstructed 3D image volumes from end-diastole CINE MRI. From each CINE short axis time stack, an image of the heart at end diastole was extracted to create a 3D volume image stack. Image extraction was performed using a custom MATLAB image processing code. The volume stacks were then segmented using the open-source Seg3D software (SCI Institute, University of Utah, SLC UT). The volume stack was segmented semi-automatically by inserting seed points along the edge of each slice. After that, the segmentation was manually modified to remove any flaws or artifacts. A binary mask volume of the completed segmentation was exported for further analysis. The segmentations were then isotropically resampled and converted to meshes using the open software ShapeWorks. In order to align the shapes, the meshes were centered and rigidly aligned to a representative reference sample selected from the population. The reference sample is selected by first computing the mean (average) mesh, then selecting the sample closest to that mean (i.e., medoid). The rigid alignment was done by calculating the transformations only using the RV meshes of the population due to their complex shapes. These transformations were then applied to the RV and the LVW meshes. The average edge length (given as mean ± std in mm) of the right ventricle meshes was .8224 ± .3987, left ventricle wall meshes was .9438 ± .3399, the IVS meshes .5196 ± .4047, and the contours 21.469 ± 26.205.

### 3.3 Shape model construction

We used ShapeWorks, an open-source software that implements the particle-based entropy optimization (Cates et al., 2007; Cates et al., 2017) described in Section 2.1. We modified the optimization with the proposed cost function (Eq. 4) to support multi-organ anatomies with shared boundaries. First, the shared boundary surface and contour were extracted for both the datasets before building shape models using the tool described in Section 2.2. During the extraction process, Laplacian smoothing was performed as per step 3 mentioned in Section 2.2 for 30 iterations with relaxation parameter set to 1. Figure 2 shows an example output for one sample of the cardiac biventricular dataset and Figure 3D shows example output for the peanut dataset. For the peanut dataset, a shape model was built using 512 particles for each shape (sphere and ellipsoid), and 64 particles each for the shared boundary surface and contour. For the cardiac dataset, a shape model was built using 512 particles for the RV and LVW, and 64 particles were used for the IVS surface and contour. We also generated a multi-organ anatomy shape model for the cardiac dataset without performing the shared boundary extraction and optimization as a baseline model for comparison. This shape model was generated using the optimization cost function specified in Eq 3 (already a part of ShapeWorks) and will be referred to as the multiple-domain shape model.

## 4 Results and discussions

### 4.1 Synthetic peanut dataset

**FIGURE 4:**
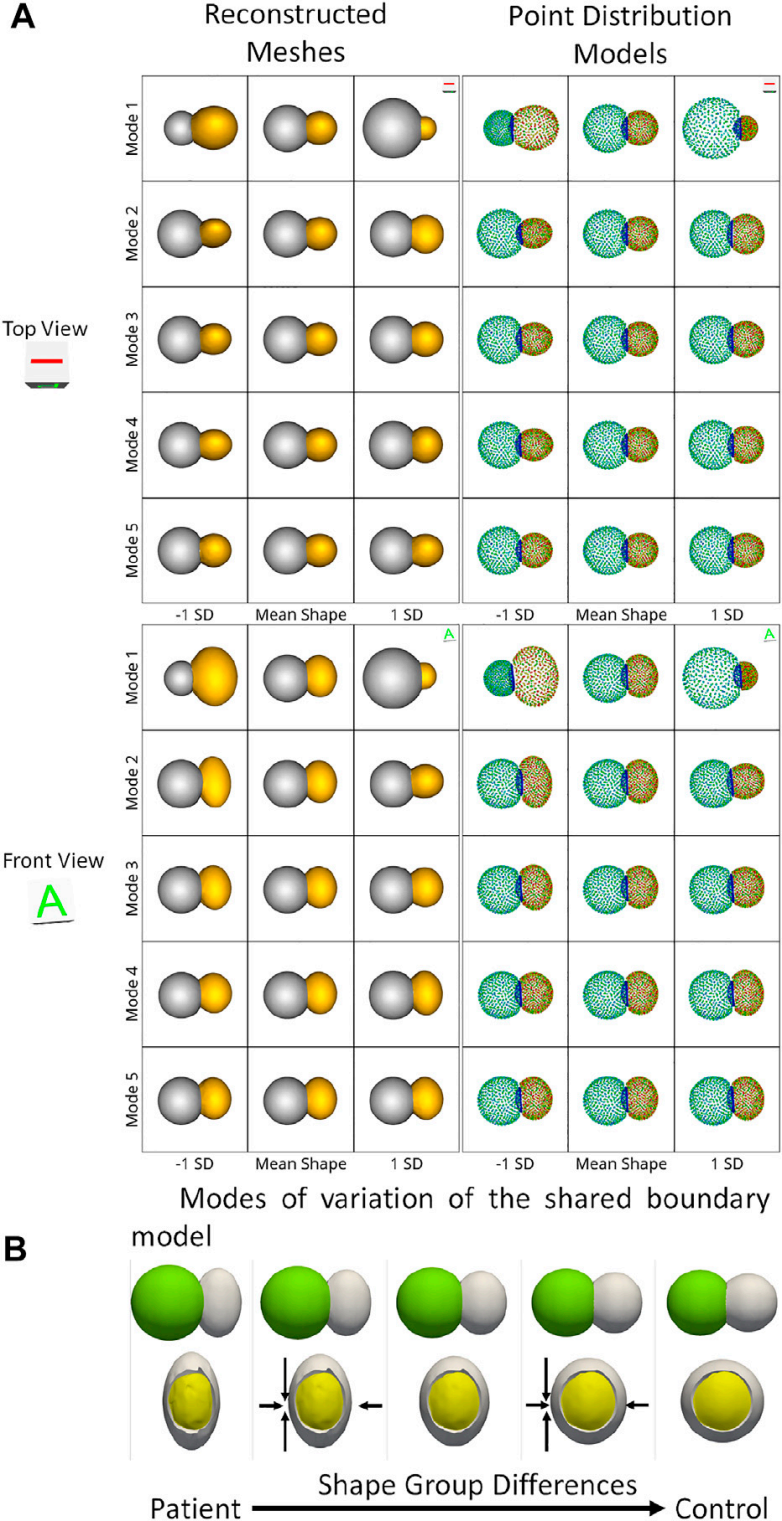
**(A)** Two viewpoints of the point distribution models and the reconstructed meshes of the modes of variations of the peanut dataset discovered by the shared boundary model. **(B)** Group-level shape differences observed by the shared boundary shape model. The arrows indicate the direction of shape change, the yellow mesh represents the extracted shared boundary, and the white represents one of the shapes of the peanut.

We use the peanut dataset as proof of concept. Figures 3C, D show the extracted shared boundary and contour for a control sample with two spheres and a patient sample with a sphere and ellipsoid. A shape model was then generated using the proposed optimization. We use PCA to simplify the complexity of the high-dimensional correspondence model while identifying the patterns learned by the PSM. Using PCA, we rank the independent modes of shape variation according to the proportion of variance that is explained (measured by eigenvalues) to the overall variance. The modes that account for the most form variability are called the dominant modes. The generated shape model was used to identify the group-level shape differences shown in Figures 4A, B. The shape model correctly identified the relative increase and decrease in size as modes of variation while appropriately representing the shared boundary surface and contour. It can be seen from the particle distribution models shown in Figure 4A that the proposed shared boundary optimization ensures that the particles from one object do not penetrate other objects while visualizing the modes of variations. From Figure 4B, we can see that the shared boundary models correctly identified partial ellipsoids as the shared surface in the case of pathology samples and partial spheres in the case of control samples as group-level shape difference. The observations made using the synthetic peanut dataset shared boundary model have successfully showcased the proof of concept and the feasibility of implementing the proposed tools.

### 4.2 Cardiac biventricular dataset

**FIGURE 5:**
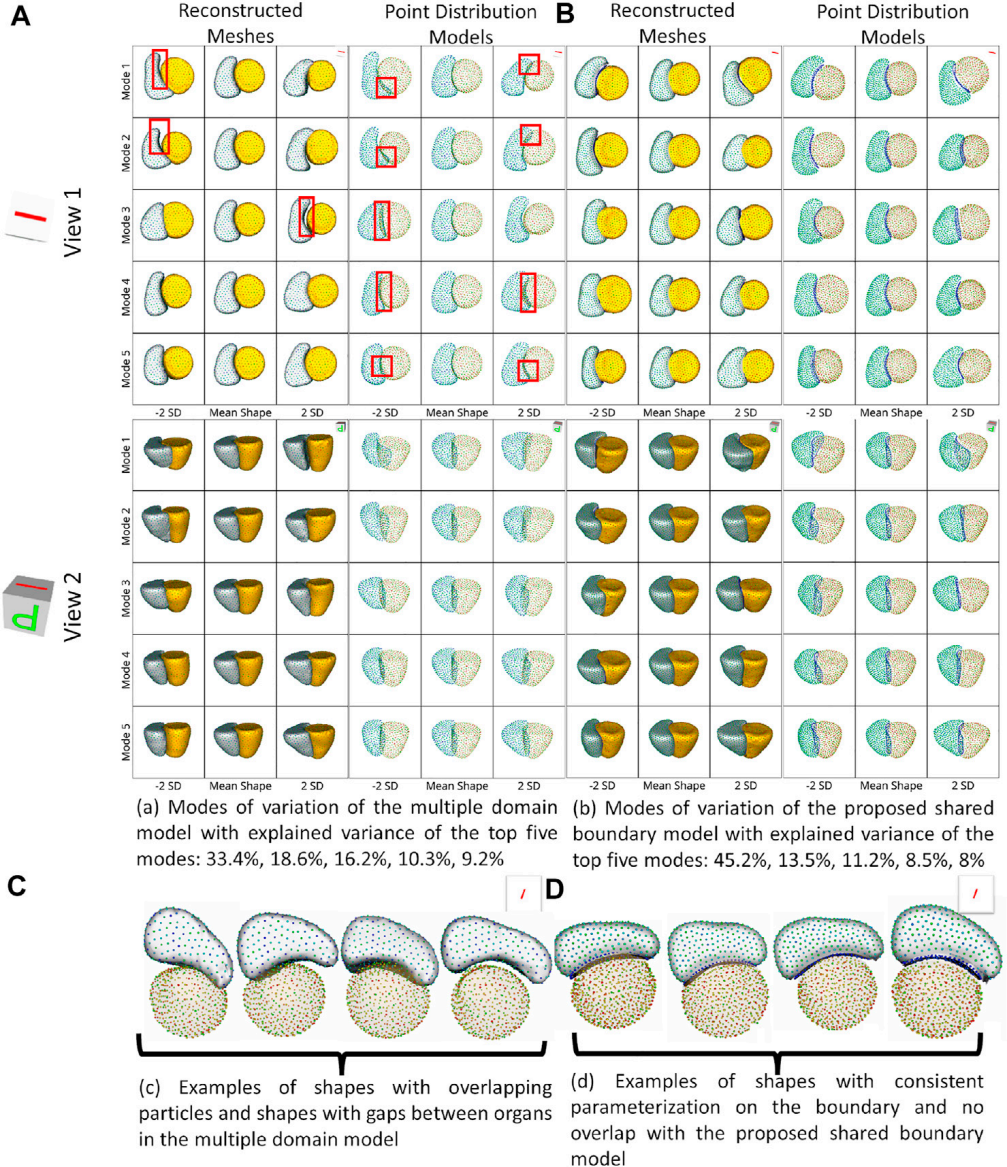
Two different views of the same reconstructed meshes and the point distribution models of the modes of variations discovered by **(A)** multiple domains model and **(B)** the proposed shared boundary model. The red boxes indicate shape modeling inconsistencies of multiple domain model - particle overlap and gaps between organs. **(C)** Examples of shapes with overlapping particles and shapes with gaps between organs in the multiple domains model and **(D)** examples of shapes with consistent parameterization in the proposed shared boundary model.

For the cardiac biventricular dataset, we compare the following two models - shared boundary and multiple domains. Both shape models are assessed on their capability to identify the underlying morphometric variations. Like the peanut dataset, PCA was used to identify the modes of variations identified by the shape models. Figure 5 shows the five dominant modes of variation discovered by the multiple domains and shared boundary model for the cardiac dataset. As multiple domain models lack the explicit parameterization of the shared boundary surface between the LVW and RV, the modes of variations show anatomical inconsistencies visible in Figures 5A, C. The particles penetrate the adjacent organs (indicated with red boxes) in the identified modes of variations rendering the learned statistics of the data clinically irrelevant. On the other hand, the shared boundary model produces clinically useful modes of variation with a consistent representation of the IVS, i.e., the shared boundary surface. The multiple domain model also generated modes of variations with that introduced gaps between the LVW and RV. This anatomical inconsistency is seen with the multiple domains shape model because the model does not consider the interactions between the two organs and fails to account for the joint statistics of the shared surface in the optimization process. In contrast, the proposed method directly models the joint statistics.

**FIGURE 6:**
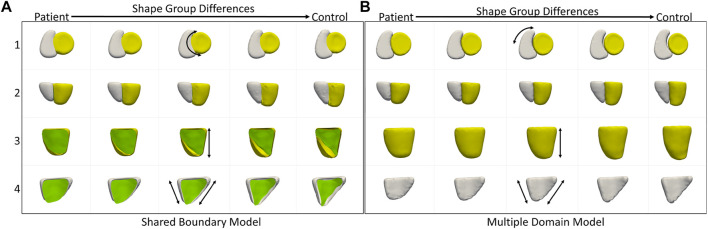
Group shape differences identified by **(A)** the proposed shared boundary and **(B)** the multiple domains model. Each row shows the samples from a different view to visualize the differences. The black arrows indicate the direction of the variation of shape.

We also compared the performance of the shared boundary model and the multiple domain model for the cardiac dataset to identify the group-level differences between pathology and controls. Figure 6A shows the group differences identified by the shared boundary model, and Figure 6B shows multiple domains. There is a marked difference in the curvature of IVS of the healthy group as compared to the patient group as identified by the shared boundary model in the first row of Figure 6A. Multiple domain model group differences show anatomical inconsistencies as the shared surface is not explicitly modeled. The anatomical inconsistencies are in the form of a gap introduced between the two organs, which is visible in the first row of Figure 6. The shared boundary removed the anatomical inconsistencies and correctly identified the shape difference describing the pathology and control group. In contrast, the multiple domains model identifies size differences correctly but fails to model the curvature of the IVS as there is no explicit representation of the IVS in the model.

### 4.3 Multi-level analysis of morphology and alignment

Statistical shape modeling aims to identify subtle variations within a population. The data acquisition process plays an essential role in determining data quality. Due to manual and system errors, the image acquisition process can introduce pose variations that result in the variation of the relative position of the organs in the anatomy. Most modeling pipelines rely on iterative closed point alignment (Arun et al., 1987; Besl and McKay, 1992) to rigidly align the cohort of shapes in the population. However, these alignment techniques cannot robustly eliminate variations in the relative pose. Therefore, we need tools that identify the pose variations and separate them from clinically relevant shape variations of the multi-organ anatomy.

**FIGURE 7:**
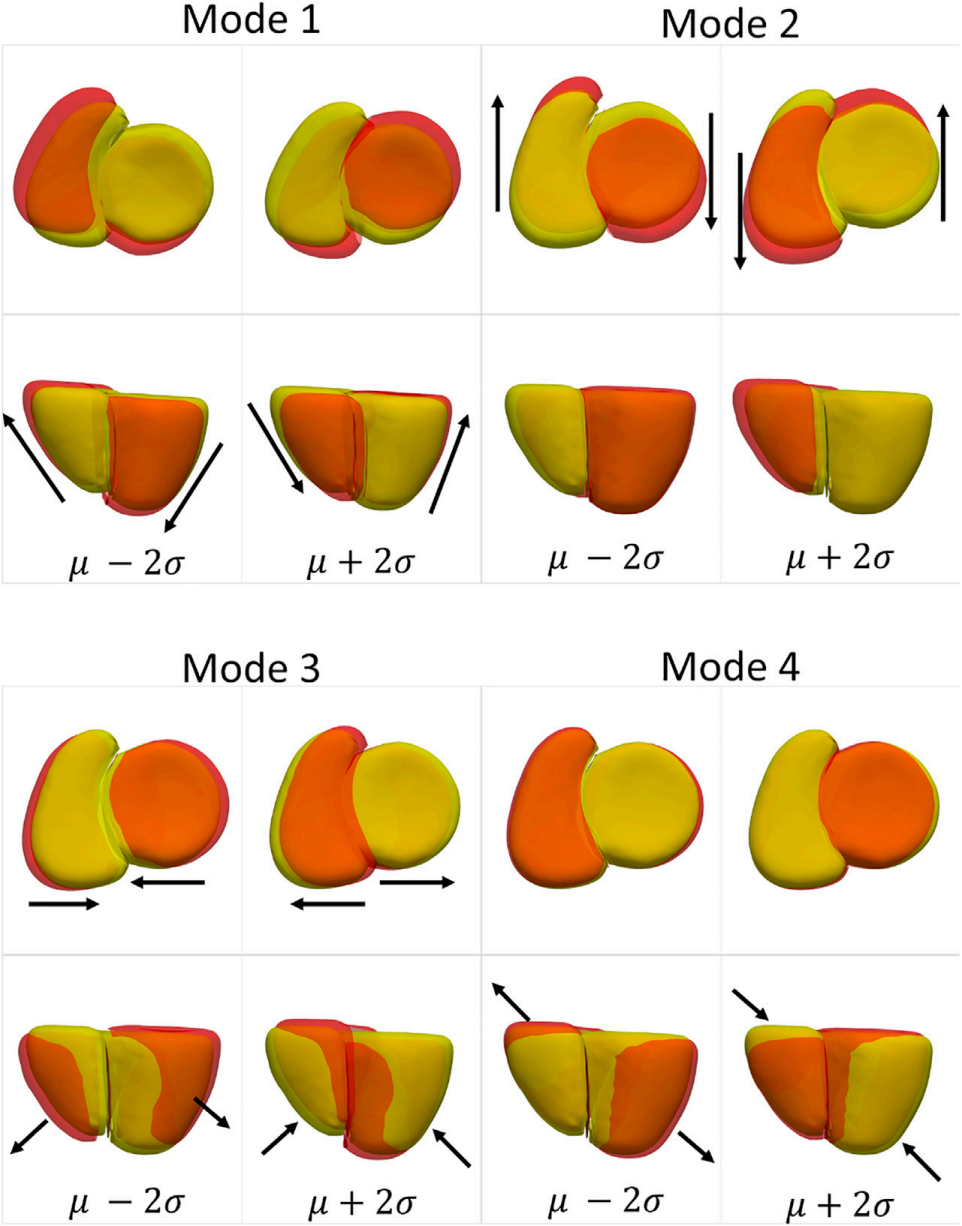
Alignment variations identified using multi-level component analysis for the cardiac dataset. The surface meshes in yellow represent the mean reconstructed shape, and the surface meshes in red represent the shapes at *μ*+2*σ* and *μ*−2*σ*. The black arrows indicate the direction of the variation of the pose.

**FIGURE 8:**
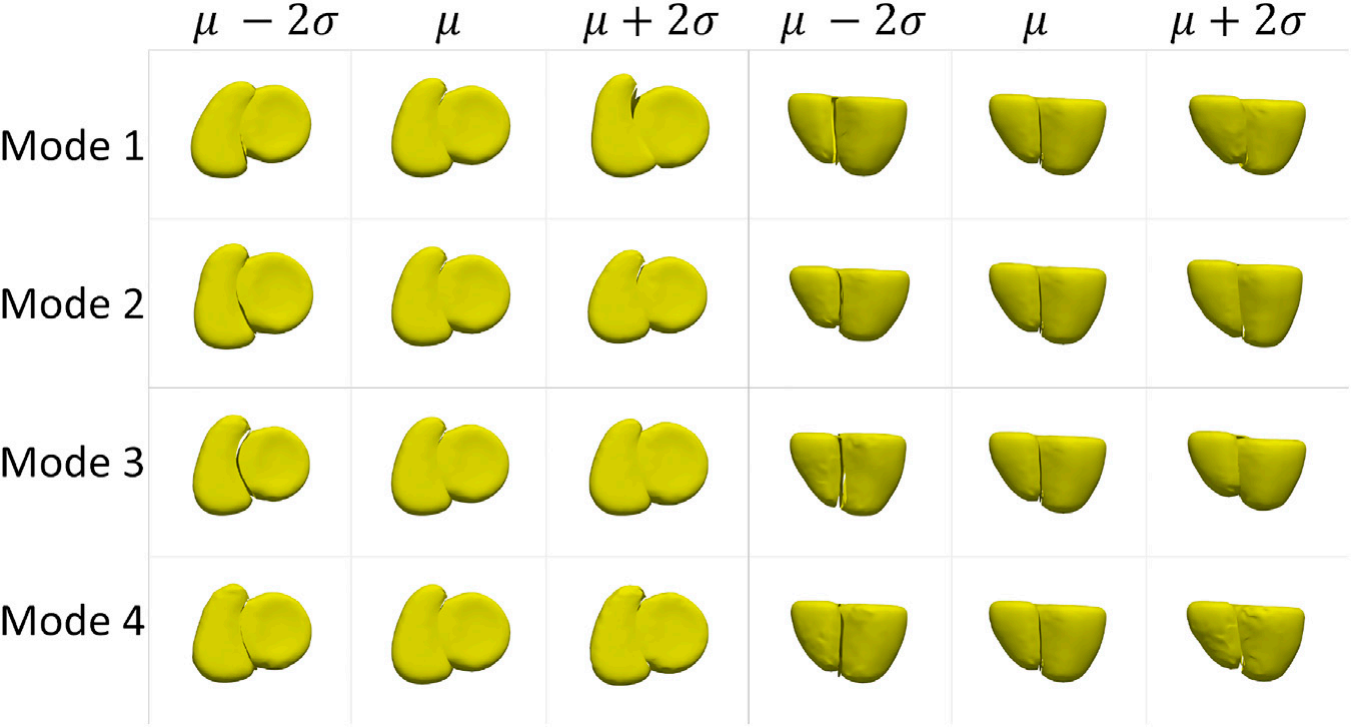
Shape variations identified using multi-level analysis for the cardiac dataset visualized from two different views.

The cardiac biventricular dataset is challenging as it contains some misalignments that could not be resolved by using rigid alignment in the pre-processing step. Hence, the modes of variation identified by using PCA for the shared boundary shape model and multiple domain model include variations in alignment and shape entangled together. The entangled observations can be seen in the first mode shown in Figures 5A, B where the right ventricle moves around the left ventricle wall. Such observations render the learned statistics clinically insignificant as these variations do not naturally occur in the anatomy. In order to mitigate the problem of entangled mode of variations, we use the multi-level component analysis (MLCA) technique (Timmerman, 2006). MLCA is an extension to PCA, where the analysis is done at different levels in which the data is observed. PCA is done on the joint shape space for the shape model having multiple organs under consideration. MLCA, on the other hand, applies PCA to capture the individual subspace of each organ under consideration that encodes the within-organ shape variations across the population and the between-organ subspace capturing the relative alignment variations across the population. Thus, applying this multi-level analysis technique helps disentangle the mode of variations into shape variations and pose (relative positioning of the organs) variations, which was not seen otherwise using PCA only. Figure 7 shows the top four dominant modes of pose variations where the arrows indicate the direction of the movement of the organs. Figure 8 shows the top four dominant modes of shape variations discovered by multi-level analysis. Comparing the disentangled shape variations in Figure 8 with the PCA modes of variations in Figure 5B, we can see that the two organs no longer showcase translation or rotation with respect to each other.

### 4.4 Data imbalance

In order to study statistically significant geometric group differences learned by the shared boundary model, we performed linear discrimination of variation. The linear discrimination between the two groups is defined as the difference vector between the particle-wise mean shapes of the two groups. The shape of each subject is then mapped/projected onto this difference vector by taking the dot product between the subject-specific shape representation (the particle correspondences) and this difference vector. The mapping results in a single scalar value (or a “shape-based score”) that places subject-specific anatomy on a group-based shape difference that is statistically derived from the shape population. The particle-wise mean shape for the cardiac patients is set as -1, and controls are set as 1. The mappings of all the other subjects are then similarly normalized relative to these values, giving a shape distribution of individual members of the population relative to the mean shapes of their respective groups. A univariate Gaussian distribution is then fitted to the normalized mapping of each group to define the probability density function of the shape scores for each group.

**FIGURE 9:**
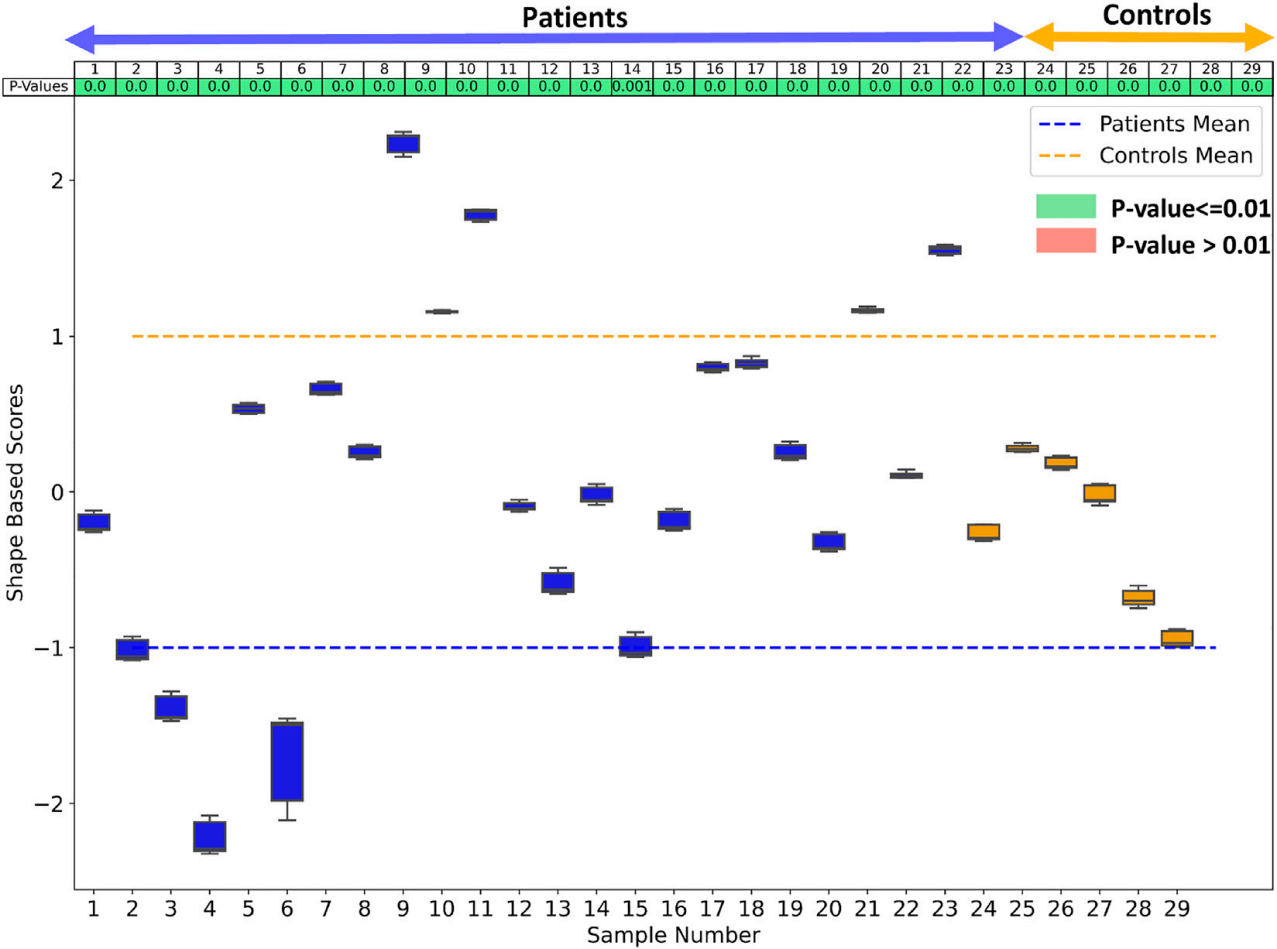
The box whisker plot shows the distribution of the shape-based scores for each sample from ten different shape models generated to study the effect of data imbalance. The table at the top of the plot shows the *p*-values of the shape-based scores.

Since the number of samples in the patient group and control group are not the same, we performed hypothesis testing to identify if the shape-based score assigned to each sample is statistically significant and agnostic to the data imbalance. We generated the shape-based scores for each sample by building a shared boundary shape model with six randomly selected samples from the patient group and all six control group samples and use these samples to generate the particle wise mean shapes and the difference vector. We then used this model to predict the particle position on the remaining samples without optimizing the shape model again. The predicted particle positions for all samples are then mapped to the difference vector to generate the shape based scores. This experiment was repeated ten times, and a shape-based score was generated for all the samples for each experiment. The shape-based scores from the experiment were then compared to those shape-based scores generated by using the shape model built with the complete dataset. We use a *t*-test to test for the null hypothesis that the expected value (mean) of a sample of independent observations from the ten trials equals the given population mean, i.e., the scores generated using the complete dataset. Figure 9 shows the box-and-whisker plot of the distribution of scores of each sample obtained from the experiment, and the table above the color indicates the *p*-values. We select the alpha value to be .01. Hence, if the *p*-values are smaller than .01, the null hypothesis holds (shown in green), and if the *p*-value is greater than .01, we can reject the null hypothesis and assume that the scores are affected by the imbalance (shown in red). As mentioned previously, the cardiac biventricular dataset is challenging and the misalignments could not be resolved with rigid alignments and the variability in scale is also high. Due to these conditions, the shape-based score for the samples are not centered around the means of patients and controls set at -1 and 1. Although it can be seen from box-whisker plots of Figure 9 that the imbalance does not affect the shape-based scores, the study of group shape differences and pathological changes can benefit from the addition of more samples and better shape alignment strategies for the biventricular database.

### 4.5 Clinical importance of shared boundary SSM

The observations from the shared boundary model confirm what has been observed in the cardiology literature: a decrease in interventricular septal curvature during prolonged right ventricular dysfunction (Marcu et al., 2006; Kochav et al., 2015; Addetia et al., 2018; Mauger et al., 2019). A healthy heart has a significant pressure gradient between the right and left ventricles (Dawes et al., 2017; Morgan et al., 2018). However, in many cardiac diseases, the pressure gradient dissipates because right ventricular pressure increases (Kochav et al., 2015). As the pressure increases, a distortion occurs at the interventricular septum, and the original septal curvature matching the left ventricular becomes flattened. This signifies the structural remodeling that occurs with severe cardiac pathologies (Farrar et al., 2016; Addetia et al., 2018).

### 4.6 Limitations

For the shared boundary extraction tool from Section 2.2, the threshold used in step 2 needs to be tuned for all the samples with high variability in the dataset. As the threshold is changed, the shape of the shared surface changes. Hence, the shared boundary extraction mechanism of the proposed tool has to be robust and needs to be improved such that the extraction is carried out based on the statistics of the entire population rather than operating on a sample level. The extracted shared boundary surfaces and contours can also showcase a very high level of variability compared to the rest of the organs, making it challenging to produce a stable shape model with meaningful modes of variations. Hence, we cannot rely on the Gaussian assumption for generating compact PDMs. In the future, the proposed PSM optimization method from Section 2.3 could be modified to incorporate non-linear shape variations for a compact and generalizable model.

## 5 Conclusion

We demonstrated that our approach preserves the integrity of the multiple-organ PDM while offering a reliable and consistent representation of the shared boundary. The unique shape changes of the IVS that are not captured when modeling the ventricles alone were demonstrated using our method on a cardiac biventricular dataset. The initial structural changes of the heart are an adaptation to overcome changes in cardiac physiology secondary to various pathologies. Prolonged exposure to these pathological changes results in chronic maladaptations that increase morbidity, and mortality (Leary et al., 2012). Patients often do not have symptoms of cardiac disease, such as shortness of breath or decreased exercise tolerance, at this stage because of the initially compensatory changes in cardiac function (Dreyfuss et al., 2004). However, structural changes, such as the IVS curvature change, are frequently visible and simple to spot. Therefore, IVS curvature changes could be used as an early identification tool to detect abnormalities before the patient develops symptoms. Shape analysis also has other clinical advantages, including being non-invasive. The current gold standard approach for assessing cardiac pressure differences is *via* invasive cardiac catheterization, which puts the patient at significant procedural risk for a diagnostic test. In conclusion, our novel approach for extracting and generating shape models of multi-organ anatomy with shared boundaries could pave the way for using statistical shape modeling from non-invasive imaging as a powerful diagnostic tool.

## Data Availability

The datasets presented in this article are not readily available because Patient data cannot be shared. Requests to access the datasets should be directed to krithika.iyer@utah.edu.

## References

[B1] Addetia K. Maffessanti F. Muraru D. Singh A. Surkova E. Mor-Avi V. (2018). Morphologic analysis of the normal right ventricle using three-dimensional echocardiography–derived curvature indices. J. Am. Soc. Echocardiogr. 31, 614–623. 10.1016/j.echo.2017.12.009 29402505 PMC5936650

[B2] Alraddadi A. (2021). Literature review of anatomical variations: Clinical significance, identification approach, and teaching strategies. Cureus 13, e14451. 10.7759/cureus.14451 33996311 PMC8117423

[B3] Andriacchi T. P. Koo S. Scanlan S. F. (2009). Gait mechanics influence healthy cartilage morphology and osteoarthritis of the knee. J. Bone Jt. Surg. Am. volume 91, 95–101. 10.2106/jbjs.h.01408 PMC2663350 19182033

[B4] Arun K. S. Huang T. S. Blostein S. D. (1987). “Least-squares fitting of two 3-d point sets,” in IEEE Transactions on pattern analysis and machine intelligence, 698–700. 10.1109/tpami.1987.4767965 21869429

[B5] Bartsch R. P. Liu K. K. Bashan A. Ivanov P. C. (2015). Network physiology: How organ systems dynamically interact. PloS one 10, e0142143. 10.1371/journal.pone.0142143 26555073 PMC4640580

[B6] Besl P. J. McKay N. D. (1992). “Method for registration of 3-d shapes,” in Sensor fusion IV: Control paradigms and data structures (Boston, MA: United States), 1611, 586–606.

[B7] Borghi A. Rodriguez Florez N. Ruggiero F. James G. O’Hara J. Ong J. (2020). A population-specific material model for sagittal craniosynostosis to predict surgical shape outcomes. Biomechanics Model. Mechanobiol. 19, 1319–1329. 10.1007/s10237-019-01229-y PMC7424404 31571084

[B8] Cates J. Bieging E. Morris A. Gardner G. Akoum N. Kholmovski E. (2014). Computational shape models characterize shape change of the left atrium in atrial fibrillation. Clin. Med. Insights Cardiol. 8, 15710. CMC–S15710. 10.4137/cmc.s15710 PMC4559307 26380559

[B9] Cates J. Elhabian S. Whitaker R. (2017). “Shapeworks: Particle-based shape correspondence and visualization software,” in Statistical shape and deformation analysis (Elsevier), 257–298.

[B10] Cates J. Fletcher P. T. Styner M. Hazlett H. C. Whitaker R. (2008). “Particle-based shape analysis of multi-object complexes,” in International conference on medical image computing and computer-assisted intervention (Springer), 477–485. 10.1007/978-3-540-85988-8_57 PMC2753605 18979781

[B11] Cates J. Fletcher P. T. Styner M. Shenton M. Whitaker R. (2007). “Shape modeling and analysis with entropy-based particle systems,” in Biennial international conference on information processing in medical imaging (Springer), 333–345. 10.1007/978-3-540-73273-0_28 PMC2768473 17633711

[B12] Cerrolaza J. J. Picazo M. L. Humbert L. Sato Y. Rueckert D. Ballester M. Á. G. (2019). Computational anatomy for multi-organ analysis in medical imaging: A review. Med. Image Anal. 56, 44–67. 10.1016/j.media.2019.04.002 31181343

[B13] Cootes T. F. Taylor C. J. Cooper D. H. Graham J. (1995). Active shape models-their training and application. Comput. Vis. image Underst. 61, 38–59. 10.1006/cviu.1995.1004

[B14] Davies R. H. (2002). Learning shape: Optimal models for analysing natural variability. The University of Manchester United Kingdom.

[B15] Dawes T. J. W. de Marvao A. Shi W. Fletcher T. Watson G. M. J. Wharton J. (2017). Machine learning of three-dimensional right ventricular motion enables outcome prediction in pulmonary hypertension: A cardiac mr imaging study. Radiology 283, 381–390. 10.1148/radiol.2016161315 28092203 PMC5398374

[B16] Dreyfuss P. Dreyer S. J. Cole A. Mayo K. (2004). Sacroiliac joint pain. JAAOS-Journal Am. Acad. Orthop. Surg. 12, 255–265. 10.5435/00124635-200407000-00006 15473677

[B17] Durrleman S. Prastawa M. Charon N. Korenberg J. R. Joshi S. Gerig G. (2014). Morphometry of anatomical shape complexes with dense deformations and sparse parameters. NeuroImage 101, 35–49. 10.1016/j.neuroimage.2014.06.043 24973601 PMC4871626

[B18] Faber B. G. Bredbenner T. Baird D. Gregory J. Saunders F. Giuraniuc C. (2020). Subregional statistical shape modelling identifies lesser trochanter size as a possible risk factor for radiographic hip osteoarthritis, a cross-sectional analysis from the osteoporotic fractures in men study. Osteoarthr. Cartil. 28, 1071–1078. 10.1016/j.joca.2020.04.011 PMC7387228 32387760

[B19] Farrar G. Suinesiaputra A. Gilbert K. Perry J. C. Hegde S. Marsden A. (2016). Atlas-based ventricular shape analysis for understanding congenital heart disease. Prog. Pediatr. Cardiol. 43, 61–69. 10.1016/j.ppedcard.2016.07.010 28082823 PMC5222611

[B20] Field D. A. (1988). Laplacian smoothing and delaunay triangulations. Commun. Appl. Numer. methods 4, 709–712. 10.1002/cnm.1630040603

[B21] Goparaju A. Iyer K. Bone A. Hu N. Henninger H. B. Anderson A. E. (2022). Benchmarking off-the-shelf statistical shape modeling tools in clinical applications. Med. Image Anal. 76, 102271. 10.1016/j.media.2021.102271 34974213 PMC8792348

[B22] Heitz G. Rohlfing T. Maurer C. R. Jr (2005). “Statistical shape model generation using nonrigid deformation of a template mesh,” in Medical imaging 2005: Image processing (San Diego, CA: United States), 5747, 1411–1421.

[B23] Iyer K. Morris A. Zenger B. Karnath K. Orkild B. A. Korshak O. (2022). Statistical shape modeling of biventricular anatomy with shared boundaries. arXiv preprint arXiv:2209.02706. 10.1007/978-3-031-23443-9_28 PMC10103081 37067883

[B24] Jacobson A. Panozzo D. (2018). libigl: A simple C++ geometry processing library. Available at: https://libigl.github.io/ .

[B25] Jesse M. K. Kleck C. Williams A. Petersen B. Glueck D. Lind K. (2017). 3d morphometric analysis of normal sacroiliac joints: A new classification of surface shape variation and the potential implications in pain syndromes. Pain Physician 20, E701–E709. 28727714

[B26] Kochav J. Simprini L. Weinsaft J. W. (2015). Imaging of the right heart—Ct and cmr. Echocardiography 32, S53–S68. 10.1111/echo.12212 25244072

[B27] Krol Z. Skadlubowicz P. Hefti F. Krieg A. H. (2013). Virtual reconstruction of pelvic tumor defects based on a gender-specific statistical shape model. Comput. aided Surg. 18, 142–153. 10.3109/10929088.2013.777973 23488562

[B28] Leary P. J. Kurtz C. E. Hough C. L. Waiss M.-P. Ralph D. D. Sheehan F. H. (2012). Three-dimensional analysis of right ventricular shape and function in pulmonary hypertension. Pulm. Circ. 2, 34–40. 10.4103/2045-8932.94828 22558518 PMC3342747

[B29] Lenz A. L. Krähenbühl N. Peterson A. C. Lisonbee R. J. Hintermann B. Saltzman C. L. (2021). Statistical shape modeling of the talocrural joint using a hybrid multi-articulation joint approach. Sci. Rep. 11, 7314–14. 10.1038/s41598-021-86567-7 33795729 PMC8016855

[B30] Li G. Yin J. Gao J. Cheng T. S. Pavlos N. J. Zhang C. (2013). Subchondral bone in osteoarthritis: Insight into risk factors and microstructural changes. Arthritis Res. Ther. 15, 223–312. 10.1186/ar4405 24321104 PMC4061721

[B31] Marcu C. B. Beek A. M. Rossum A. C. V. (2006). Cardiovascular magnetic resonance imaging for the assessment of right heart involvement in cardiac and pulmonary disease. Heart, Lung Circulation 15, 362–370. 10.1016/j.hlc.2006.08.003 17045525

[B32] Marrouche N. F. Wilber D. Hindricks G. Jais P. Akoum N. Marchlinski F. (2014). Association of atrial tissue fibrosis identified by delayed enhancement mri and atrial fibrillation catheter ablation: The decaaf study. Jama 311, 498–506. 10.1001/jama.2014.3 24496537

[B33] Mauger C. Gilbert K. Lee A. M. Sanghvi M. M. Aung N. Fung K. (2019). Right ventricular shape and function: Cardiovascular magnetic resonance reference morphology and biventricular risk factor morphometrics in UK biobank. J. Cardiovasc. Magnetic Reson. 21, 41–13. 10.1186/s12968-019-0551-6 PMC6637624 31315625

[B34] McInerney T. Terzopoulos D. (1996). “Deformable models in medical image analysis,” in Proceedings of the workshop on mathematical methods in biomedical image analysis (IEEE), 171–180.

[B35] Morgan A. E. Zhang Y. Tartibi M. Goldburg S. Kim J. J. Nguyen T. D. (2018). Ischemic mitral regurgitation: Abnormal strain overestimates nonviable myocardium. Ann. Thorac. Surg. 105, 1754–1761. 10.1016/j.athoracsur.2018.01.00510.1016/j.athoracsur.2018.01.005 29391146 PMC6005393

[B36] Orkild B. A. Zenger B. Iyer K. Rupp L. C. Ibrahim M. M. Khashani A. G. (2022). All roads lead to rome: Diverse etiologies of tricuspid regurgitation create a predictable constellation of right ventricular shape changes. Front. Physiology 13, 908552. 10.3389/fphys.2022.908552 PMC9291517 35860653

[B37] Paulsen R. Larsen R. Nielsen C. Laugesen S. Ersbøll B. (2002). “Building and testing a statistical shape model of the human ear canal,” in International conference on medical image computing and computer-assisted intervention (Springer), 373–380.

[B38] Postacchini R. Trasimeni G. Ripani F. Sessa P. Perotti S. Postacchini F. (2017). Morphometric anatomical and ct study of the human adult sacroiliac region. Surg. Radiologic Anat. 39, 85–94. 10.1007/s00276-016-1703-0 27324173

[B39] Samson C. Blanc-Féraud L. Aubert G. Zerubia J. (2000). A level set model for image classification. Int. J. Comput. Vis. 40, 187–197. 10.1023/a:1008183109594

[B40] Sanfilippo A. J. Abascal V. M. Sheehan M. Oertel L. B. Harrigan P. Hughes R. A. (1990). Atrial enlargement as a consequence of atrial fibrillation. a prospective echocardiographic study. Circulation 82, 792–797. 10.1161/01.cir.82.3.792 2144217

[B41] Smith H. (2021). Anatomical variation and clinical diagnosis. diagnostics 11, 247. 10.3390/diagnostics11020247 33562542 PMC7915783

[B42] Styner M. Oguz I. Xu S. Brechbühler C. Pantazis D. Levitt J. J. (2006). Framework for the statistical shape analysis of brain structures using spharm-pdm. insight J. 242, 242–250. 10.54294/owxzil PMC3062073 21941375

[B43] Timmerman M. E. (2006). Multilevel component analysis. Br. J. Math. Stat. Psychol. 59, 301–320. 10.1348/000711005x67599 17067414

[B44] Uetani M. Tateyama T. Kohara S. Tanaka H. Han X.-H. Kanasaki S. (2015). Statistical shape model of the liver and its application to computer-aided diagnosis of liver cirrhosis. Electr. Eng. Jpn. 190, 37–45. 10.1002/eej.22668

[B45] Valette S. Chassery J. M. Prost R. (2008). Generic remeshing of 3d triangular meshes with metric-dependent discrete voronoi diagrams. IEEE Trans. Vis. Comput. Graph. 14, 369–381. 10.1109/tvcg.2007.70430 18192716

